# Trends of Online Search of COVID-19 Related Terms in Cyprus

**DOI:** 10.3390/epidemiologia2010004

**Published:** 2021-01-20

**Authors:** Marios Anastasiou, Katerina Pantavou, Anneza Yiallourou, Stefanos Bonovas, Georgios K. Nikolopoulos

**Affiliations:** 1 Independent Scholar, Nicosia 2740, Cyprus; anastasioumariosam@gmail.com; 2 Medical School, University of Cyprus, Nicosia 2029, Cyprus; pantavou.katerina@ucy.ac.cy (K.P.); yiallourou.anneza@ucy.ac.cy (A.Y.); 3 Department of Biomedical Sciences, Humanitas University, 20090 Milan, Italy; stefanos.bonovas@hunimed.eu; 4 Humanitas Clinical and Research Center-IRCCS, 20089 Milan, Italy

**Keywords:** COVID-19, pandemic, google trends, search interest, information seeking, internet behavior

## Abstract

Knowledge of trends in web searches provides useful information for various purposes, including responses to public health emergencies. This work aims to analyze the popularity of internet search queries for Coronavirus Disease 2019 (COVID-19) and COVID-19 symptoms in Cyprus. Query data for the term Coronavirus were retrieved from Google Trends website between 19 January and 30 June 2020. The study focused on Cyprus and the four most populated cities: Nicosia, Limassol, Larnaca, and Paphos. COVID-19 symptoms including fever, cough, sore throat, shortness of breath, and myalgia were considered in the analysis. Daily and weekly search volumes were described, and their correlation with the evolution of the COVID-19 pandemic and important announcements or events were examined. Three periods of interest peaks were identified in Cyprus. The highest interest in COVID-19-related terms was found in the city of Paphos. The most popular symptoms were fever and cough, and the symptom with the highest increase in popularity was myalgia. At the beginning of the pandemic, the search volume of COVID-19 grew substantially when governments, major organizations, and high-profile figures, globally and locally, made important announcements regarding COVID-19. Health authorities in Cyprus and elsewhere could benefit from constantly monitoring the online interest of the population in order to get timely information that could be used in public health planning and response.

## 1. Introduction

Knowing what is popular among the public can help health authorities monitor information spread that could be associated with disease transmission, progression, and control. Google Trends (GT) is a very popular, free online tool that provides information on public interest in various topics [1]. Several studies [1,2,3,4] have focused on public interest using data from GT and found a relationship between GT and health data, including those on Coronavirus Disease 2019 (COVID-19). For instance, Liu et al. [2] analyzed the United States (US) audience’s GT data and showed that when high profile figures endorsed the therapeutic use of chloroquine and hydroxychloroquine for COVID-19, the relative search queries for buying these products increased. Another research group [4] studied GT data until late February 2020 from US, United Kingdom (UK), Australia, Canada, New Zealand, and Ireland showing that the peak of interest in COVID-19 was on 31 January 2020 in all countries. However, the speed of interest growth, the response time, and the duration of public attention differed across them.

The aim of this work was to examine the online interest of Cypriots in COVID-19 and its symptoms during the first months of the COVID-19 pandemic. The methods and results could be used in serious infectious disease outbreaks and other public health crises for information management and understanding, for facilitating awareness, for making predictions, and for implementing effective public health measures.

## 2. Materials and Methods

The online interest of the Cypriot population was examined based on Google Trends [5], which is a tool for the analysis of the popularity of top search queries in Google. The main advantages are the anonymity of searches, and the use of revealed instead of stated preferences of Google users [6]. The methodology followed in this work was based on the framework presented in the study of Mavragani and Ochoa [1]. Interest Score (IS) was used as a metric of popularity. IS is a normalized measure of the online interest of the audience. It represents the search interest relative to the highest point on the chart for a given region and time. A value of 100 is the peak popularity for a given term. A value of 50 means that the term is half as popular. A score of 0 means that there was not enough data for this term. An IS is developed after choosing search term(s), region(s), period, and category in the GT explore website [7].

### 2.1. Area of Study

The Republic of Cyprus (capital Nicosia) is an island country in the Eastern Mediterranean Sea. It is a member state of the European Union and a very popular tourist destination. The population was 875,900 (government-controlled area) in 2018 [8], while 3,938,625 tourist arrivals were recorded in the same year [9]. Most households had internet access in 2020 (92.8% or 285,051 out of 307,246) [10]. The majority of internet users (16–74 years old-*n* = 656,378) are females (51.1%, *n* = 338,227) and in the age group of 25–54 years (59%, *n* = 386,388). A slightly higher percentage of females than males (51.6%, *n* = 265,019 versus 48.4%, *n* = 249,005) search for information about products or services, while slightly more males than females (50.6%, *n* = 237,712 versus 49.4%, *n* = 231,732) read online news, newspapers, or magazines [10].

### 2.2. Search Strategy, Data, and Analysis

The required search term was added on the GT explore website [7] defining region (i.e., Cyprus) and time period. The latter was set from 19 January 2020 (first day when IS > 0 in Cyprus) until 30 June 2020 (end of first pandemic wave in Cyprus-before the second surge of cases in July 2020) [11]. All categories were selected to capture the total interest of people in Cyprus about all aspects of COVID-19 (e.g., health, economic, phycological, etc.). Three different cases were explored: (a) Overall, (b) by city, and (c) per symptom. Several steps of the procedure (i.e., search terms, region) were different depending on the case.

Various search terms were tested to identify the most popular one. Among Coronavirus, COVID, COVID-19, COVID 19, SARS, and the Greek terms Κορονοιος, Κορονοιός, Κορονοϊός, Κορωνοϊός the most popular was Coronavirus. Thus, the popularity of the term Coronavirus was examined considering the overall region of Cyprus. Then, the Coronavirus search term was examined for each of the four most populated cities of Cyprus, i.e., Nicosia, Limassol, Larnaca, and Paphos. The topic of “Virus”, instead of the default, which is “Search Term”, was used. Topics include terms that share the same concept in any language, and therefore, they capture a larger scale of the total real online interest [12]. The search terms Fever (Medical Condition), Cough (Disease), Sore throat (Topic), Shortness of breath (Disease), and Myalgia (Topic) were considered for the region of Cyprus. The examined symptoms were those which had significant online interest in the selected time period, and were also related to COVID-19, based on the World Health Organization (WHO) [13].

IS was downloaded directly from GT. The Google search queries used to create IS were collected from the Web, Image, News, Google Shopping, and YouTube Search.

Global and local announcements and events related to COVID-19 were monitored and searched in Google. These were about governmental, major organizations, and high-profile figures’ statements regarding the COVID-19 pandemic that may have affected public attention and interest in seeking information on the internet.

Line graphs were used to present the trend of IS over time and mean, median values, standard deviation, and interquartile range were used to describe IS per symptom.

## 3. Results

### 3.1. Google Trends

**Figure 1:**
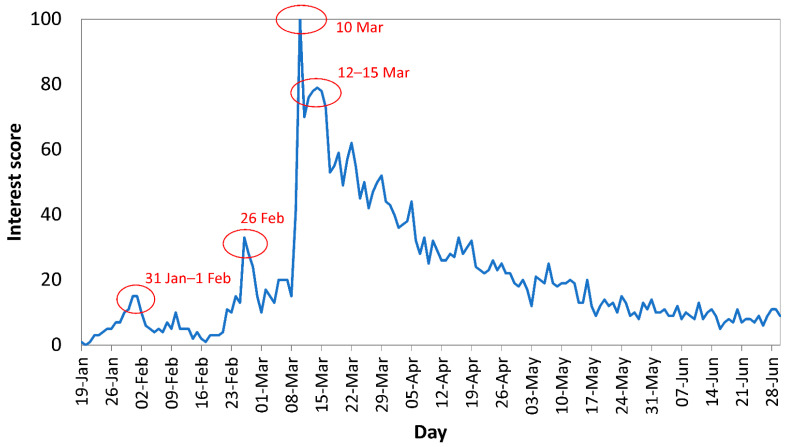
Interest Score in Google search for the search query Coronavirus between 19 January and 30 June 2020 in Cyprus.

The trend of IS over time (Figure 1) showed that the online search interest in COVID-19 increased from 19 to 31 of January 2020 (IS = 15). A second IS peak (IS = 33) occurred on 26 February 2020 followed by a third peak (maximum) on 10 March (IS = 100) and from 12 to 15 March 2020 (IS range 76 to 79). IS was low after 17 May 2020, ranging between 5 and 15.

**Figure 2:**
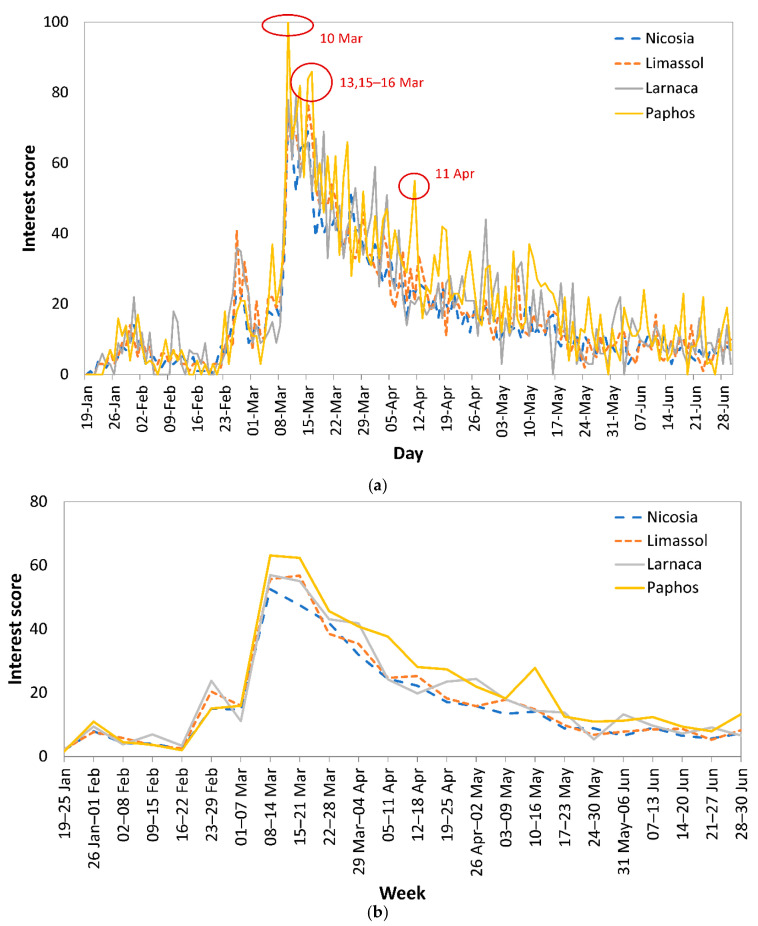
(**a**) Interest Score trend and (**b**) weekly average of Interest Score over the time period 19 January to 30 June 2020 across Nicosia, Limassol, Larnaca, and Paphos, based on the search query Coronavirus.

Analysis by city showed that online search interest in COVID-19 in all four cities, Nicosia, Limassol, Larnaca, and Paphos, followed similar trends (Figure 2a). Nevertheless, IS was higher in Paphos on 10 March 2020 (IS = 100), 13 March 2020 (IS = 82), 15 March 2020 (IS = 84), 16 March 2020 (IS = 86), and 11 April 2020 (IS = 55). The trend of the weekly average of IS (Sunday to Saturday; Figure 2b) confirmed that during weeks with maximum IS (8–14 and 15–21 March 2020), the highest value was recorded in Paphos. The highest IS was in Paphos, and the lowest in Nicosia, until 30 June 2020 (Figure 2b).

**Figure 3:**
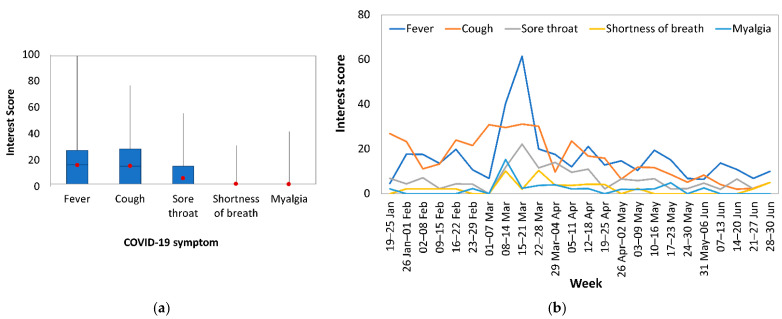
(**a**) Boxplot and average value (red dot) of Interest Score, and (**b**) weekly average of Interest Score of COVID-19 symptoms between 19 January and 30 June of 2020 in Cyprus.

Figure 3a shows IS quartiles of five symptoms of COVID-19 (fever, cough, sore throat, shortness of breath, and myalgia). Overall, the main symptoms of interest were cough and fever. Although the trend of weekly average of IS (Figure 3b) showed differences between symptoms, the IS of all symptoms increased around 8 March to 21 March 2020. A second peak of IS was found for fever in 12–18 April 2020 and 10–16 May 2020, and for cough in 5–11 April 2020.

**Figure 4:**
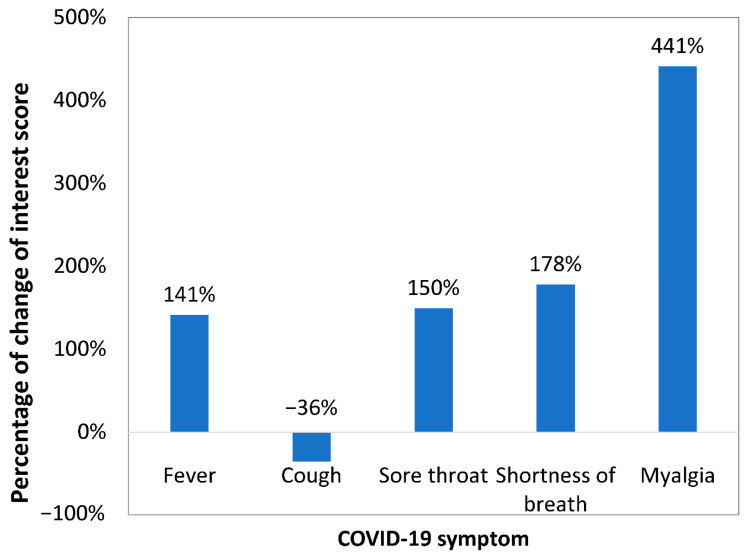
Percentage of change of Interest Score between 19 January–8 March 2020 and 9 March–30 June 2020 in Cyprus.

The popularity of symptoms changed before and after the announcement of the first COVID-19 case in Cyprus (9 March 2020) [14]. This change was higher for sore throat, shortness of breath, and myalgia, which had a 150%, 178%, and 441% increase, respectively (Figure 4).

### 3.2. Interest Score and Announcements or Events

**Figure 5:**
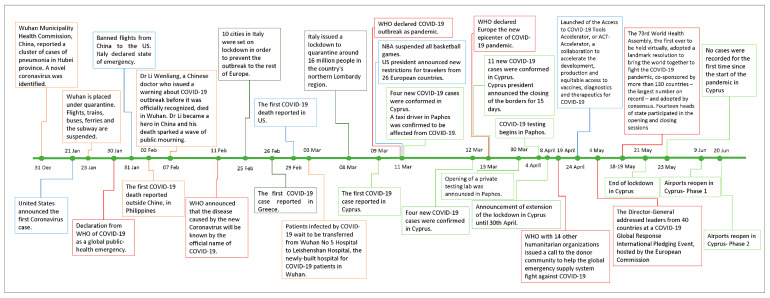
Timeline of COVID-19 pandemic; events and announcements probably affecting popularity of the Coronavirus search query in Google search in Cyprus.

A research of major events and announcements around IS peaks (Figure 5) showed that the first IS peak (31 January 2020) occurred on the day after (30 January 2020) the WHO’s declaration of COVID-19 as a global public-health emergency [15]. Moreover, on 31 January 2020, the Italian government declared a state of emergency over COVID-19, [16] and the US president announced a ban of most foreign nationals from getting into the US if they were in China within the prior two weeks [17]. The second IS peak (26 February 2020) was found the day after the announcement related to the increase of confirmed new cases and the lockdown in 10 cities in Italy [18] (25 February 2020). On the same day (26 February 2020), the first COVID-19 case was confirmed in Greece, a country closely related to Cyprus, sharing the same language and cultural characteristics [19]. The third IS peak (10 March 2020 and 12–15 March 2020) was observed close to the dates that the first COVID-19 cases were confirmed in Cyprus (9 March 2020) [14,20,21,22,23] and the announcement of the President of Cyprus about closing country’s borders for 15 days [24]. IS was steadily low close to the major lift of the strict lockdown (21 May 2020 [25]) and until the end of June 2020, when airports in Cyprus reopened (20 June 2020) [11] and the first wave of COVID-19 ended.

The research on events in Cyprus around the IS peaks in Paphos (13 and 15–16 March 2020 and 11 April 2020; Figure 2) showed that on 11 March 2020, a taxi driver in Paphos was diagnosed with COVID-19 [20]. On 30 March 2020, the opening of a private testing lab was announced [26], on 4 April 2020, extensive COVID-19 testing began in Paphos [27], while on 8 April 2020, the President of Cyprus announced an extension of the lockdown, which was originally set until 10 April 2020 [28].

## 4. Discussion

Google data clearly indicated days of high internet search interest in COVID-19-related terms in Cyprus. Search interest peaked the same or the next day of announcements by governments, authorities, organizations, or high-profile figures in COVID-19 pandemic. Nevertheless, the duration of interest was short. Variations of search interest across cities were also observed. The highest interest in COVID-19 was found in Paphos and the lowest in Nicosia. The increased popularity in Paphos was possibly due to local events and the high diagnosis rate in the city early in the first COVID-19 pandemic wave in Cyprus. Search interest in all symptoms of COVID-19 that were examined was high at the beginning of the COVID-19 pandemic in Cyprus. The interest in sore throat, shortness of breath, and myalgia continued to increase during the first pandemic wave, while the interest in cough and fever significantly declined. The interest in shortness of breath and especially in myalgia increased greatly after COVID-19 reached Cyprus.

Public response and duration of interest toward COVID-19 have been found to vary across settings [4]. Thus, studies in different countries including Cyprus contribute to expanding knowledge base and to between-countries comparisons. The results of this work, however, are in agreement with those of previous studies. The search interest of the population in other settings was also affected by official announcements [2]. Moreover, the duration of interest was short and varying across sub-regions in US, UK, Canada, Ireland, Australia, New Zealand, and Taiwan [3,4]. Interest differences across regions could be attributed to variations in the population residing in those areas, in local economic parameters, the transportation system, in tourism development, and in numbers of confirmed cases.

This study has some limitations that must be taken under consideration. IS is a metric generated by Google Trends that takes a random sample and not the total number of Google searches [29]. Moreover, the sample characteristics (i.e., age, gender) are unknown. However, Google Trends have been useful in predicting the onset of seasonal influenza and other infectious disease epidemics [30,31,32]. In addition, the search query used in this study was general, covering all COVID-19-related terms (e.g., vaccines, therapies), and universal, permitting comparisons with similar data in different countries, while the search volume used was sufficient to provide evidence [33] in regions with high internet access. Finally, this was a descriptive study aiming to provide the authorities with some useful information that could be used in their response to the COVID-19 pandemic. Further studies should examine the potential relationship of Google Trends with health data or the willingness of people to get a vaccine, as well the ability of Google Trends to predict an increase in COVID-19 incidence or in cases of other infectious diseases in Cyprus. Of note, a lag time between COVID-19 search interest and daily new cases (12 days) and new deaths (19 days) was reported in the US [34]. Similarly, a lag time (10–14 days) between the peaks of search interest and of COVID-19 incidence was observed in China [35].

In conclusion, this work showed that Google Trends can be used to identify periods of high search interest in COVID-19. The period with the highest IS for Coronavirus in Cyprus was between 12 and 15 March 2020, around the time the first cases of COVID-19 were diagnosed, and the city with the overall highest IS was Paphos. The symptoms with the highest IS were cough and fever, while shortness of breath and especially myalgia showed a substantial increase in interest over time. These patterns can be useful to health authorities in the context of their response to the future COVID-19 waves or to other outbreaks.

## Data Availability

The data presented in this study are available on request from the corresponding author.
